# Recent Work on the Demyelinating Diseases
*Based on a communication to the West Country Physicians' Club, April 1958.


**Published:** 1959-01

**Authors:** N. S. Alcock

**Affiliations:** Consultant Neurologist, Exeter.


### RECENT WORK ON THE DEMYELINATING DISEASES*

BY

N. S. ALCOCK, M.D., F.R.C.P.

*Consultant Neurologist, Exeter.*

The outstanding problem in neurology during the last twenty to thirty years has been the cause of disseminated sclerosis. The solution may be a little nearer now and this review covers some of the developments during this period.

Just over twenty years ago J. R. M. Innes came to the National Hospital for Nervous Diseases and showed us some intriguing pictures of lambs with sway-back and suggested that here was a demyelinating disease which could be prevented by giving the ewes copper. Then, during the war, four workers studying sway-back developed themselves some curious symptoms resembling disseminated sclerosis. This naturally stimulated a considerable amount of work into the possible connection between copper—or other trace elements—and disseminated. To determine the normal ranges the copper levels in other patients in the neurological wards were examined. This was how the abnormal levels in Wilson's disease were found and the cause of this disorder accidentally established: to remind you—in Wilson's disease (hepato-lenticular degeneration) it is suggested that excessive copper is absorbed from the intestine, perhaps by the failure of some mechanism which normally inhibits it. Then, instead of being converted within 24 hours to caeruloplasmin, it remains loosely attached to albumen and is therefore presumably free to be deposited in liver and basal ganglia. Chemical diagnosis depends on demonstrating a high copper excretion in the urine or, probably easier and more reliable, a low caeruloplasmin level in blood. We are now able to complete the picture by treating these patients with B.A.L. or penicillamide. The connection between Wilson's disease and sway-back is a tenuous one—there is too much copper in the one and too little in the other. There are also many problems in sway-back awaiting solution; the way it occurs on fields which have been heavily limed, the fact that unaffected ewes may have equally low blood copper, and the difficulty in correlating the pathological picture and clinical signs, are unexplained.

The next step in the story is work on the neurological complications of the exanthemata. It has long been recognized that the encephalomyelitides which occasionally complicate measles, chicken pox and rubella all look very similar. Originally it was suggested that these were a direct virus infection; then, when this proved unacceptable, it was suggested that the illness fanned into activity a virus lying dormant in the central nervous system, an explanation which seems much too ingenious to be true. It was then shown that a pathologically identical condition could be produced by the injection of sterile normal brain tissue together with an adjuvant consisting of killed tubercle bacilli; the substances which produce this change consist of a polysaccharide and polypeptide linked to mycolic acid. Some recent work has however suggested that several closely allied substances can produce the same effects. An interesting extension of this work has been the production of a similar pathological changes in the thyroid by the injection of thyroid tissue with an adjuvant, and in the same way changes have been produced in suprarenals and peripheral nerves.

On the clinical side it has been shown that although there is considerable variation in the latent periods of these neurological complications, the average latent period is shortest in the encephalitis group and longest in the polyradiculitis—in fact approximately double that for the encephalitides—and that this order holds true for each of

* Based on a communication to the West Country Physicians' Club, April 1958.

the diseases even though the average latent period varies between the different in[illegible] tions. This can be seen from Miller's figures (published in *Modern Trend*[illegible] *Neurology*, Williams, D. 1957):

Average latent period in days of neurological complications following measles, varicella and rubella.

| | Measles | Varicella | Rubella |
|---|---|---|---|
| Encephalomyelitis | 4·7 | 6·3 | 3·8 |
| Myelitis | 5·5 | 8·25 | 6·7 |
| Polyradiculitis | 9 | 11·25 | 8·7 |

Miller also brings into this group not only these complications of the exanthe[illegible] but also the isolated brachial plexus palsies after serum and T.A.B. innoculati[illegible] the neuralgic amyotrophy described by Parsonage and Turner (1948), acute haem[illegible] hagic leuco-encephalitis and the neurological catastrophes that happen occasion[illegible] with anti-rabies treatment and prophylaxis. These cases are, however, comparati[illegible] rare and the work on them seems to have rather branched off from the main prob[illegible] of the cause or mechanism of disseminated sclerosis. Even here there has per[illegible] been a little progress. Firstly a little more is known of the composition of myeli[illegible] has been shown that there is an appreciable day to day turnover of myelin lipids. [illegible] the metabolism of myelin is obviously slow, perhaps connected with the slow evolu[illegible] of the process in disseminated sclerosis. Sphingomyelin and cerebrosides ap[illegible] during the first year of life and the biochemical development continues until se[illegible] maturity. Thus while cerebroside values gradually increase, sphingomyelin, [illegible] the first few months, steadily decreases so that the cerebroside/sphingomyelin [illegible] rises throughout childhood and adolescence. It is interesting to speculate whe[illegible] this could have any connection with the fact that disseminated so rarely, if ever, be[illegible] before adolescence.

The recent work on tissue culture is also interesting. Lumsden has shown th[illegible] tissue culture, cells particularly oligodendrocytes are not the static structures se[illegible] stained preparations, but move and elongate and contract in a surprisingly m[illegible] way and may well be intimately concerned with myelination. This work, how[illegible] has not yet had any practical application.

Returning to the question of allergy there has been an interesting obser[illegible] published from Oxford. In the Oxford centre for tuberculous meningitis am[illegible] the treatments used, is the injection of tuberculin into the C.S.F., and it has [illegible] shown that the response to this varies with the state of the Mantoux reaction. [illegible] response is remarkably consistent and once the result of the Mantoux test was k[illegible] the character and intensity of the intrathecal response could be predicted with re[illegible] able certainty. It could, however, be considerably modified by giving cortiso[illegible] the same time as the tuberculin. This led to the suggestion, that it might be ben[illegible] to patients with disseminated sclerosis to be given intrathecal tuberculin; and i[illegible] found that the meninges in patients with disseminated sclerosis react differently [illegible] the normal—at least in the Mantoux positive group. In the Mantoux negative, [illegible] the normal, no definite reaction was obtained. In the others, however, the res[illegible] not only lacked consistency and regularity but was also different in character, [illegible] much nearer to that obtained when the normal is modified by cortisone. So it [illegible] possible that the normal immunological processes must be disordered in some [illegible] in fact the reaction could well be explained if one postulates that there is a[illegible] some substance with a cortisone-like effect.

It is interesting in this connection to speculate whether this could have an[illegible] nection with the fact that even the most acute disseminated fails to make any res[illegible] to cortisone whereas many of the acute encephalomyelitis cases react well to i[illegible]

Lastly, intrathecal tuberculin does seem to be beneficial to some patients with disseminated sclerosis. But we must accept this with caution, for even in the last twenty years there have been a good many different theories and treatments for disseminated sclerosis which have come and gone: venous thrombosis and vaccines, spherules, spirochaetes and diphtheroids, fever therapy and arsenic have all had their protagonists and although at the moment allergy or some disturbance in the antibody-antigen reaction seems to be in the ascendant, this is a long way from being proven; that is why this paper is entitled "recent work" and not "recent advances".

### SELECTED REFERENCES

Greenfield, J. G., Blackwood, W., McMenemey, W. H., Meyer, A. and Norman, R. M. (1958). *Neuropathology*, London.

*Lancet*—leader on Wilson Disease. (1957). **1**, 976.

McAlpine, D., Compston, N. D. and Lumsden, C. E. (1955). *Multiple Sclerosis*. Edinburgh and London.

Miller, H. G., Stanton, J. B. and Gibbons, J. L. (1956). *Quart. J. Med.* **25**, 428.

Parsonage, M. J. and Turner, J. W. A. (1948). *Lancet* (1). 973.

Smith, Honor V., Espir, M. L. E., Whitty, C. W. M. and Russell, W. R. J. (1957). *Neurol. Neurosurg. Psychiat.* **20**, 1.

Williams, D. (1957). *Modern Trends in Neurology*. London, Second series.

